# The development of descending projections from the brainstem to the spinal cord in the fetal sheep

**DOI:** 10.1186/1471-2202-8-40

**Published:** 2007-06-18

**Authors:** Elaine M Stockx, Colin R Anderson, Susan M Murphy, Ian RC Cooke, Philip J Berger

**Affiliations:** 1 Ritchie Centre for Baby Health Research, Monash Institute of Medical Research, Monash University, Clayton, Victoria, 3168, Australia; 2 Department of Anatomy and Cell Biology, Melbourne University, Melbourne, Victoria, 3010, Australia; 3 Burnet Institute, Melbourne, Victoria, 3010, Australia

## Abstract

**Background:**

Although the fetal sheep is a favoured model for studying the ontogeny of physiological control systems, there are no descriptions of the timing of arrival of the projections of supraspinal origin that regulate somatic and visceral function. In the early development of birds and mammals, spontaneous motor activity is generated within spinal circuits, but as development proceeds, a distinct change occurs in spontaneous motor patterns that is dependent on the presence of intact, descending inputs to the spinal cord. In the fetal sheep, this change occurs at approximately 65 days gestation (G65), so we therefore hypothesised that spinally-projecting axons from the neurons responsible for transforming fetal behaviour must arrive at the spinal cord level shortly before G65. Accordingly we aimed to identify the brainstem neurons that send projections to the spinal cord in the mature sheep fetus at G140 (term = G147) with retrograde tracing, and thus to establish whether any projections from the brainstem were absent from the spinal cord at G55, an age prior to the marked change in fetal motor activity has occurred.

**Results:**

At G140, CTB labelled cells were found within and around nuclei in the reticular formation of the medulla and pons, within the vestibular nucleus, raphe complex, red nucleus, and the nucleus of the solitary tract. This pattern of labelling is similar to that previously reported in other species. The distribution of CTB labelled neurons in the G55 fetus was similar to that of the G140 fetus.

**Conclusion:**

The brainstem nuclei that contain neurons which project axons to the spinal cord in the fetal sheep are the same as in other mammalian species. All projections present in the mature fetus at G140 have already arrived at the spinal cord by approximately one third of the way through gestation. The demonstration that the neurons responsible for transforming fetal behaviour in early ontogeny have already reached the spinal cord by G55, an age well before the change in motor behaviour occurs, suggests that the projections do not become fully functional until well after their arrival at the spinal cord.

## Background

Axonal inputs from the brain to the spinal cord are known to have a profound influence on the motor activity generated spontaneously during early development in the fetal sheep. All fetal and embryonic vertebrates from early in their development exhibit a cyclic pattern of motor activity consisting of alternating periods of activity followed by long lasting periods of inactivity [1-3]. A characteristic of this form of behaviour is that all skeletal body musculature tend to be activated and deactivated synchronously. Spinal cord transections performed during this developmental stage have little effect on behaviour [2,4-8]. This finding, together with the fact that the isolated spinal cord preparation displays cyclic activity [9-13] demonstrates that this pattern of activity is generated within the spinal cord.

Later in ontogeny, cyclic behaviour is replaced by motor activity patterns in which muscles display longer, continuous periods of activity and different muscle groups are activated independently [1,3,14,15]. Once this more mature pattern of behaviour is established it is dramatically affected by spinal cord transection which causes behaviour to revert to the cyclic form seen in early development [16]. This observation strongly points to an essential role for projections from supraspinal neurons in the transition of behaviour from the cyclic pattern to the more mature form. In the fetal sheep this transition in behaviour occurs at approximately G65 [3]. As supraspinal projections are responsible for this transition we hypothesised that the neurons that bring it about have axonal projections that reach the spinal cord just prior to G65.

The sequence of arrival of supraspinal inputs at the spinal cord level has been intensively investigated in a number of animal species, including the chick [17-22], rat [23-26], opossum [27-33], reptile [34], amphibian [35-42], and fish [43,44]. While the timing of arrival of supraspinal inputs is known for many species, existing neuroanatomical studies have not been directly related to the transition in motor activity that occurs during early ontogeny. In addition to establishing which neurons may be responsible for this transition in the sheep, determining the origin of supraspinal projections would assist in the interpretation of previous work in which the sheep model was used to study physiological systems that are controlled by neuronal projections from the brainstem to spinal cord; for example swallowing [45,46] and gut motility [47].

This study had two aims. The first was to identify all the brainstem nuclei containing neurons that send descending projections to the spinal cord in the fetal sheep. The second was to examine the timing with which descending projections from the brainstem reach the spinal cord in the fetal sheep with a view to establishing which projection(s) might be responsible for the transition in motor activity that occurs during early development. The principle underlying the study is that sets of projections that are present at G140 but absent in fetuses at G55 could be responsible for the transition in motor activity seen during early ontogeny. These two aims were carried out by injecting the retrograde tracer cholera toxin subunit B (CTB) containing a coloured dye marker into the spinal cord at the level of C3-C6 in fetal sheep. The location of CTB-labelled cells in the brainstem at G55 and G140 was established with the aid of a neuroanatomical atlas [48].

## Results

Visual examination of the injection site at post mortem revealed that the blue marker dye, and hence, presumably CTB, was restricted to the ventral horn in three of the four G140 fetuses studied; in the fourth, the extent of the CTB injection site was not available at postmortem as the spinal cord was damaged during dissection. Data from this individual were included in our analysis, as the distribution of labelled cells in the brainstem of this fetus was like that seen in the other 3 fetuses of this developmental age. At postmortem of G55 fetuses, the blue dye, and presumably the CTB, in all fetuses was concentrated around the site of injection, but it was found to have spread from its ventral horn into the dorsal horn and across the midline to the opposite side of the spinal cord.

**Figure 1:**
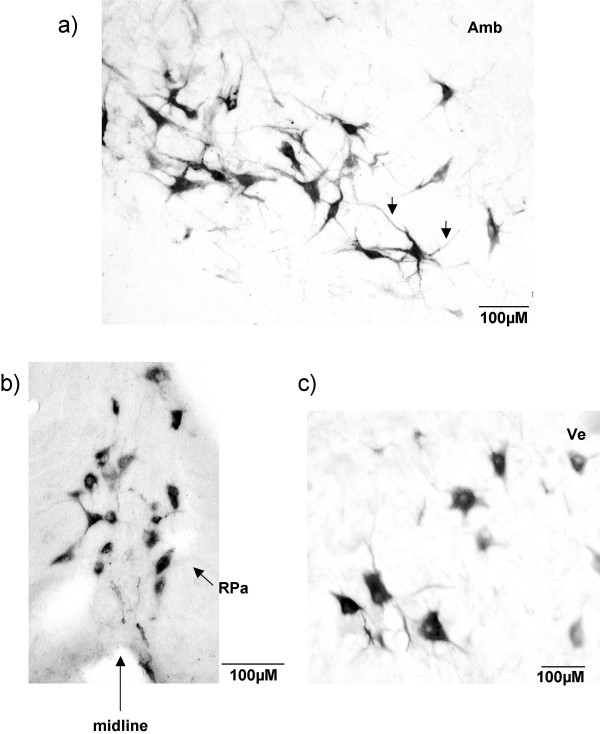
**Examples of CTB immunolabelling**. High magnification micrograph of CTB labelled cells in a G140 fetus. A) Labelled cells lie within and around the nucleus ambiguus (Amb): CTB labelled cells are of large size and have an irregular triangular shape with pronounced dendritic labelling (arrow). B) CTB labelled cells in the raphe pallidus (RPa) nucleus which spanned the ventral midline: the majority of these labelled cells were medium in size and rounded in shape. C) Labelled neuron in the vestibular nucleus (Ve): CTB labelled cells had a variety of shapes and consisted of medium to large size cells.

All brainstem nuclei containing CTB labelled neurons, and hence spinally-projecting neurons, are listed in Table 1. CTB labelled neurons (Fig. 1) were found along the entire rostro-caudal extent of the brainstem with particular concentration in the reticular nuclei of the medulla and pons in both G140 and G55 fetuses. In most cases, CTB labelling of neurons was similar ipsilateral and controlateral to the side of the tracer injection, so the line drawings facing the digitally imaged hemi-sections shown in the figures represent the labelling pattern on the side ipsilateral to the injection. Any distinct differences in the distribution of labelled CTB cells between ipsilateral and contralateral sides is described in the text below. With few exceptions, which are also described in the text, the same nuclei contained CTB labeled cells in the G55 and G140 fetuses.

**Table 1:** Location of retrogradely labelled neurons in brainstem nuclei of the fetal sheep at G140 and G55.

**Nucleus**	**Gestational Age**	
	**G140**	**G55**
"cap" around the icp		
Ambiguus nucleus		
Cells within pyramidal tract		
Cuneate nucleus		
Deep mesencephalic nucleus		
Dorsal paragigantocellular reticular nucleus		
Dorsal raphe nucleus		
Edinger-Westphal nucleus		
External cuneate nucleus		
Gigantocellular reticular nucleus		
Gracile nucleus		
Gray matter of dorsal horn		
Gray matter of ventral horn		
Intermediolateral gray		
Interstitial nucleus of the mlf		
Kölliker-Fuse nucleus		
Labelling along border edge of cuneate nucleus		
Labelling around the superior olivary complex		
Lateral reticular nucleus		
Locus coeruleus		
Medullary reticular formation (dorsal)		
Medullary reticular formation (ventral)		
Nucleus of Darkschewitsch		
Nucleus of the solitary tract		
Nucleus of the trapezoid body		
Parabrachial nucleus		
Paramedian reticular nucleus		
Parvocellular reticular nucleus		
Pontine reticular nucleus (caudal)		
Oral pontine reticular nucleus		
Raphe magnus nucleus		
Raphe obscurus nucleus		
Raphe pallidus nucleus		
Red nucleus		
Spinal trigeminal nucleus		
Subcoeruleus		
Superior collicular nucleus		
Vestibular nucleus		

= supraspinal projections within the nucleus.  = no neurons within the nuclei contain supraspinal projections.

### G140 fetus

#### Rostral spinal cord and spino-medullary junction

**Figure 2:**
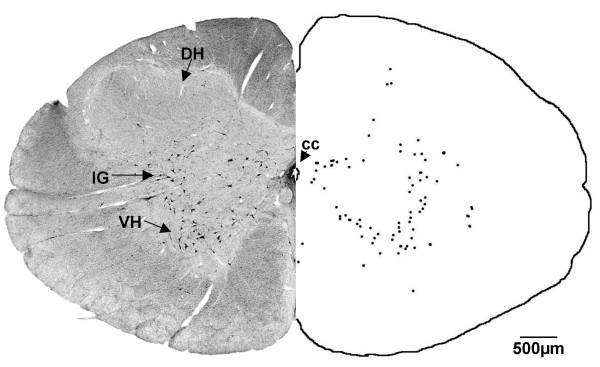
**Rostral spinal cord (G140)**. After injection of CTB into the cervical spinal cord, labelled cells were found in the rostral spinal cord, in the ventral horn (VH) and intermediolateral gray (IG). DH = dorsal horn. Both sides of the figure are ipsilateral labelling (see methods).

In the G140 fetus at the level of the rostral spinal cord, labelled neurons, varying in size, were found within the gray matter, predominantly within the ventral horn and intermediolateral gray (Fig. 2). Based on the location of the cells in the spinal cord we suggest they are propriospinal neurons with projections to more caudal spinal nuclei. There were a few labelled cells between the pyramidal tracts at the spino-medullary junction.

#### Caudal to the obex

**Figure 3:**
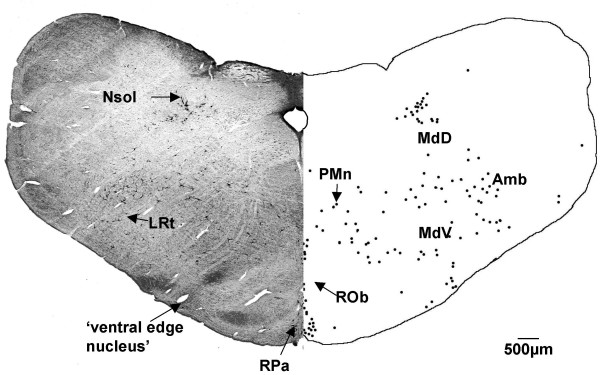
**Level before obex (G140)**. At G140, CTB labelled cells were found in the raphe pallidus (RPa), raphe obscurus (ROb), medullary reticular nucleus ventral and dorsal (MdV and MdD), nucleus of the solitary tract (Nsol), the lateral reticular nucleus (LRt), in and around the ambiguus nucleus (Amb), paramedian reticular nucleus (PMn) and in the 'ventral edge nucleus'. Both sides of the figure are ipsilateral labelling (see methods).

At a level just caudal to the obex (Fig. 3) there was labelling of cells around and within the ambiguus nucleus and the lateral reticular nucleus. These cells had irregular triangular shapes and were large in size with a number of strongly-labelled dendrites (see arrows in Fig. 1a). The medullary reticular nuclei (dorsal and ventral) contained many labelled cells, with a denser aggregation of cells ventrally, compared to the dorsal region. Labelled cells were found in the paramedian reticular nucleus and in the raphe complex, both in the raphe pallidus and raphe obscurus nuclei. The majority of cells within the raphe pallidus nucleus, which spanned the ventral midline, had a rounded shape (Fig. 1b). There was labelling of cells located immediately within and around the solitary tract. A number of cells were labelled ventral to the inferior olive and lateral reticular nucleus.

#### Level of the obex

**Figure 4:**
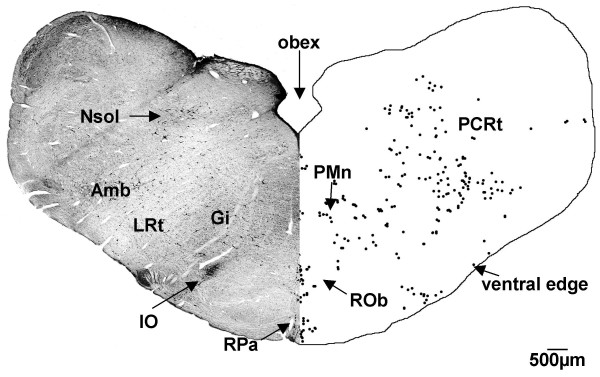
**Medulla (G140)**. CTB labelled cells were found in the medulla of the G140 sheep in the raphe pallidus (RPa), raphe obscurus (ROb), gigantocellular reticular (Gi), and parvicellular reticular (PCRt) nuclei, in the nucleus of the solitary tract (Nsol), lateral reticular nucleus (LRt), around the ambiguus nucleus (Amb), paramedian reticular nucleus (PMn), and along the ventral edge of the section. IO = inferior olive nucleus. Both sides of the figure are ipsilateral labelling (see methods).

At the level of the obex (Fig. 4) labelling continued in the raphe pallidus and raphe obscurus nuclei, in and around the ambiguus nucleus, the lateral reticular nucleus and the nucleus of the solitary tract. Labelling was present in the gigantocellular reticular and the parvicellular reticular nuclei. There was a group of labelled cells located along the border between the cuneate nucleus and the nucleus of the solitary tract. Labelled cells were also found along the ventral edge of the brainstem sections.

#### Level of the mid-medulla

**Figure 5:**
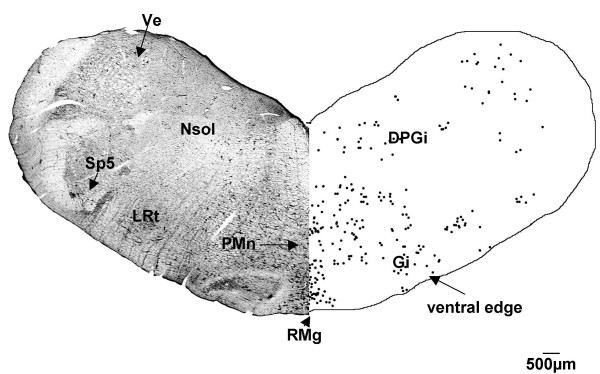
**Medulla (G140)**. In the medulla of the G140 fetal sheep, CTB labelled cells were present in the raphe magnus (RMg), gigantocellular reticular (Gi), and vestibular (Ve) nuclei, in the nucleus of the solitary tract (Nsol), spinal trigeminal (Sp5), lateral reticular (LRt), paramedian reticular (PMn), dorsal paragigantocellular (DPGi) nuclei and along the ventral edge of the brainstem section. Both sides of the figure are ipsilateral labelling (see methods).

At the level of the mid-medulla, CTB labelled cells were present in the gigantocellular reticular nucleus (Fig. 5) bilaterally. The cells were present at a much higher density ipsilateral to the side of the CTB injection. Just ventral to the gigantocellular reticular nucleus there was a large number of labelled cells within the raphe magnus nucleus. Labelled cells continued to be found in the nucleus of the solitary tract, the lateral reticular nucleus and along the ventral edge of the brainstem sections. There was a small number of CTB labelled cells in the spinal trigeminal nucleus, restricted to its ventral part. Labelled cells were observed in the caudal pole of the vestibular nucleus and in the dorsal paragigantocellular reticular nucleus.

**Figure 6:**
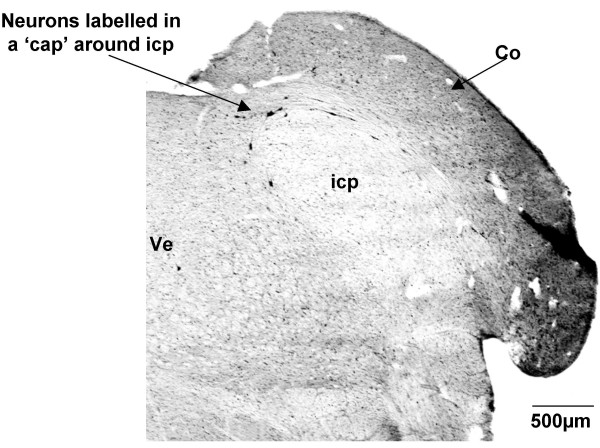
**Labelled cells around the inferior cerebellar peduncle (G140)**. CTB labelled neurons formed a 'cap' around the inferior cerebellar peduncle (icp), and also extended along the medial border of the cochlear nucleus (Co). Ve = vestibular nucleus. Both sides of the figure are ipsilateral labelling (see methods).

At this level of the medulla, CTB-labelled cells formed a 'cap' around the inferior cerebellar peduncle (Fig. 6). These neurons lay along the medial border of the cochlear nucleus and the lateral border of the vestibular nucleus and represent a nucleus that could not be defined solely on architectonic grounds or deduced from previous studies of descending projections. Based on their position, these cells are most likely part of the vestibular nucleus [48].

#### Level of the rostral medulla

**Figure 7:**
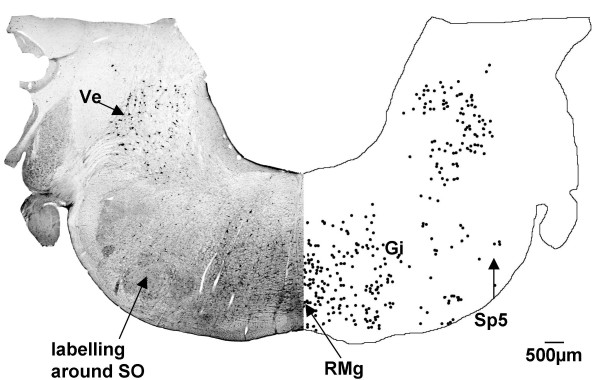
**Rostral medulla (G140)**. In the rostral medulla of the G140 sheep fetus, CTB labelled cells are found in the vestibular nucleus (Ve), gigantocellular reticular nucleus (Gi), spinal trigeminal nucleus (Sp5), raphe magnus nucleus (RMg) and around the superior olivary nucleus (SO). Both sides of the figure are ipsilateral labelling (see methods).

At this level numerous cells were labelled in the vestibular nucleus bilaterally, but with a slight ipsilateral predominance. These cells were large in size with strong dendritic labelling (Fig. 1c). Labelling continued within the gigantocellular reticular nucleus and the raphe magnus nucleus, while a small number of labelled cells were found in the spinal trigeminal nucleus still restricted to the ventral part, and around the superior olivary nucleus (Fig. 7).

#### Level of the caudal pons

**Figure 8:**
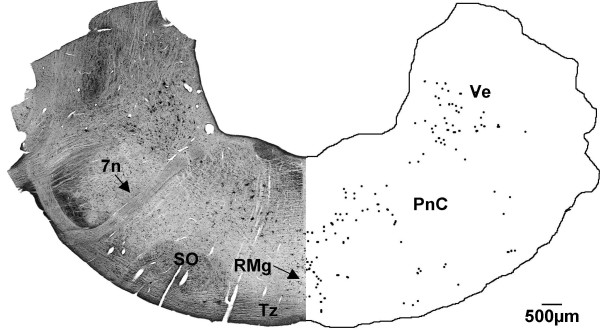
**Caudal pons (G140)**. CTB labelled cells were found in the caudal pons of the G140 sheep fetus in the raphe magnus nucleus (RMg), caudal pontine reticular nucleus (PnC), the vestibular nucleus (Ve) and the nucleus of the trapezoid body (Tz). 7n shows the facial nerve, SO = superior olivary nucleus. Both sides of the figure are ipsilateral labelling (see methods).

In the pontine region, a group of large labelled cells was observed in the caudal pontine reticular nucleus, located between the facial nerve and the midline (Fig. 8). Labelling continued in the raphe magnus and vestibular nuclei at this level. There were a few labelled cells along the ventral surface of the sections, which were presumed to be in the nucleus of the trapezoid body. A small number of labelled cells surrounded the superior olivary nucleus.

**Figure 9:**
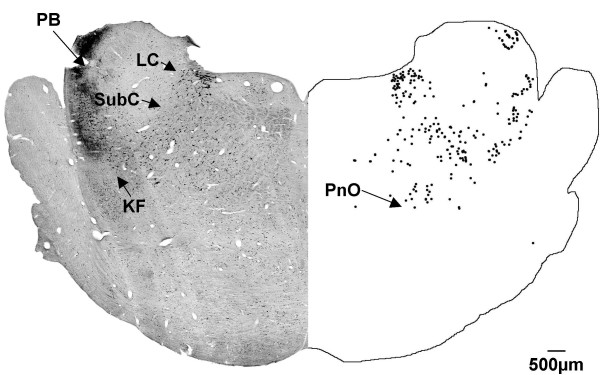
**Rostral pons (G140)**. In the rostral pons of the G140 sheep fetus, CTB labelled cells were found in the locus coeruleus (LC), and the subcoeruleus (SubC) nuclei, the oral pontine reticular nucleus (PnO), the parabrachial nucleus (PB) and the Kölliker-Fuse nucleus (KF). Both sides of the figure are ipsilateral labelling (see methods).

At a more rostral level (Fig. 9), labelled cells were found in the locus coeruleus and subcoeruleus nuclei. CTB labelled cells were also observed in the oral pontine reticular nucleus and the parabrachial region. Some labelled cells were observed below the subcoeruleus nucleus, in what is presumptively identified as the Kölliker-Fuse nucleus.

#### Level of the midbrain

At the level of the aqueduct (data not shown) there were labelled neurons in the deep mesencephalic nucleus, the oral pontine reticular nucleus, and the dorsal raphe nucleus. Labelling was observed dorsal to the aqueduct, in the location of the superior collicular nucleus.

**Figure 10:**
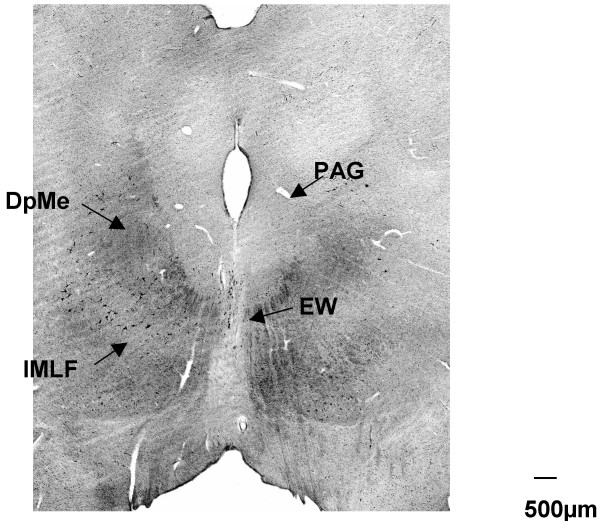
**Level at the rostral pole of the aqueduct (G140)**. Coronal section at the most rostral extent of the brainstem analysed in a G140 fetal sheep. CTB labelled cells were seen in the vicinity of the Edinger-Westphal nucleus (EW) and surrounding the periaqueductal gray. Labelled cells are found in the region between the interstitial nucleus of the medial longituidinal fasiculus (IMLF) and the deep mesencephalic nucleus (DpMe).

At the rostral pole of the aqueduct, there was labelling in the vicinity of the Edinger-Westphal nucleus (Fig. 10) and along the lateral edge of the periaqueductal gray. The nuclei to which these labelled cells belong to could not be confidently established; the most likely being the interstitial nucleus of the medial longitudinal fasciculus, but alternatively, they may belong to the cuneiform or deep mesencephalic nuclei.

Large labelled neurons were present in the red nucleus, almost entirely contralaterally, although in one fetus, labelled neurons were present bilaterally in the magnocellular region. The instance of bilateral labelling came from the fetal sheep in which the extent of the spinal injection site of CTB could not be confirmed post mortem, raising the possibility that tracer may have leaked from its site of injection into the contralateral ventral horn. However, all other aspects of CTB labelling in this fetus were similar to that seen in the other G140 fetuses where leakage of tracer was not observed.

More rostrally, there were labelled cells in the nucleus of Darkschewitsch, but these numbered only 2–3 per section (data not shown).

### G55 fetus

**Figure 11:**
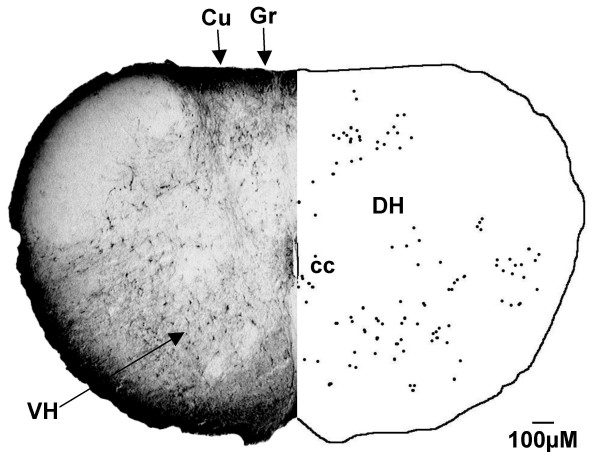
**Rostral spinal cord (G55)**. In the G55 fetus, CTB labelled cells were found in the ventral horn (VH) and dorsal horn (DH) of the spinal cord, rostral to the site of injection. Labelled cells were also observed in the gracile nucleus (Gr) and cuneate nucleus (Cu). cc = central canal. Both sides of the figure are ipsilateral labelling (see methods).

In the G55 fetus, the distribution of labelled neurons was similar to that of the G140 fetus (see Table 1) with a few exceptions, as outlined below. CTB labelled cells were observed in the gracile and cuneate nuclei (Fig. 11) at the level of the spino-medullary junction, whereas there were no labelled cells in either of these nuclei in the G140 fetus (Fig. 2).

**Figure 12:**
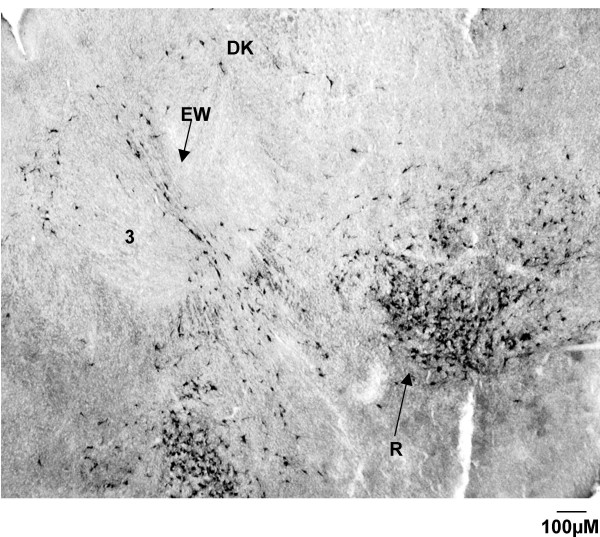
**Red nucleus (G55)**. CTB labelling in the red nucleus of a G55 fetus. Note the CTB labelling is bilateral, and forms a distinct tight cluster, with a greater density of cells towards the ventral region of the nucleus. Labelling is also observed in the nucleus of Darkschewitsch (Dk) and in the vicinity of the Edinger-Westphal nucleus (EW). 3 = oculomotor nucleus.

The group of labelled cells above the inferior cerebellar peduncle in the G140 fetus which formed a 'cap' was not present in the G55 fetus. Labelled neurons were present bilaterally in the magnocellular region of the red nucleus of the G55 fetus (Fig. 12), unlike the G140 fetus, in which the labeling was predominantly ipsilateral.

## Discussion

Physiological studies show that at approximately G60-G65 in the fetal sheep, supraspinal inputs to spinal circuits mediate a transition in motor activity from a form comprising distinct cycles of synchronous activity and inactivity to a form involving more continuous and independent activation of the muscles of the body [3,16]. Accordingly, we hypothesised that a single identifiable group, or perhaps more than one group, of neurons in the brainstem would send their descending axons to the spinal cord at an age close to G65, and that such a group of neurons would constitute a strong candidate as the mediator of the observed maturation in fetal motor activity at that time. However, we found that all brainstem nuclei that project axons to the spinal cord in the mature fetus have already done so by G55. The presence of these neurons is unlikely to reflect transneuronal labeling as CTB has previously been shown to be restricted to first order neurons [49].

Our results demonstrate that axons from all groups of spinally-projecting neurons in the brainstem reach the spinal cord of the fetal sheep well before the disappearance of the cyclic motor pattern, raising the question as to why the mature pattern of motor behaviour does not develop earlier. The axons of the neurons responsible for the transition in motor activity are unlikely to originate from higher centers of the brain, such as the diencephalon or telencephalon, as spinal projections from such areas have been shown to terminate primarily within sympathetic and parasympathetic preganglionic nuclei [50,51]. One possibility is that the axons that mediate the transition in motor activity have reached the spinal cord but have not yet provided a functional innervation of the motoneurons. Okado and Oppenheim [18] showed that supraspinal projections in the chick embryo first reach the spinal cord at E5 and the invasion of the spinal cord by the axons of brainstem neurons is largely complete by E8-E10. However, behavioural studies involving spinal cord transections show that supraspinal inputs do not play a role in embryonic motor activity until after E10 [5] or as late as E15 [7]. Some of the delay can be explained by a "waiting period" such that after the fibres of supraspinal neurons descend in the ventral and lateral funiculi there is a two day period before axons penetrate the gray matter, and a further delay of approximately one and half days before synapses form with spinal neurons [21]. Karimi-Abdolrezaee et al. [52] found that there was not only a delay in the invasion of the gray matter by corticospinal axons, but increased branching of axons and formation of synaptic contacts occurred over a period of 2 weeks once the initial invasion occurred. A waiting period appears to be a general phenomenon in the central nervous system in that there is a delay of approximately 2–3 days after the arrival of thalamocortical axons at the subplate in prenatal rats and the formation of functional thalamocortical synapses [53].

A second possible explanation is that in the fetal sheep, there is synaptic contact between the descending axons and their spinal targets well before G65, but that these synapses are not yet functional. Synaptic efficacy relies on the maturation of both the pre- and post-synaptic membrane, including the formation of concentrated acetylcholine receptors and the accumulation of synaptic vesicles [see [54] for review]. Development of synapse function, including the regulation of synaptic strength and the regulation of postsynaptic receptors, is initially dependent on the spontaneous motor activity that occurs during early ontogeny [55,56]. A third possibility is that the transition in motor activity requires synaptic input to the spinal cord from the candidate supraspinal neurons and that activity within these supraspinal neurons is itself dependent upon neural drives from other parts of the brain. Thus, the transition in motor activity may await the generation of adequate presynaptic drive to the candidate supraspinal neurons.

After injection of CTB into the rostral cervical cord of the G140 fetus, CTB labelled cells were found within identifiable nuclei in the medulla and pons, as well as throughout the reticular formation, in the vestibular nucleus, raphe complex, red nucleus, and the nucleus of the solitary tract [48]. This distribution of labelling is similar to that previously reported for retrogradely labelled, spinally projecting neurons in the chick [18], the rat [25] and in a group of 22 mammals comprising another study [57].

In the G55 fetus, CTB labelled cells were observed in the gracile and cuneate nuclei, but such cells were not found in the G140 fetus. Spinally-directed projections from these somatosensory nuclei have been demonstrated in other species [18,25,41,57]. In a number of amphibian species, and more recently, in the rat, labelling of neurons in the somatosensory nuclei only occurred if the retrograde tracer was applied to the dorsal or dorsolateral part of the spinal cord [41,58]. The presence of CTB labelled cells in the G55 fetus indicates that there was indeed leakage of tracer into parts of the dorsal horn, as also indicated by the spread of dye seen when the spinal cord was examined post mortem. The restriction of spread of the CTB tracer to the ventral horn in the G140 fetus in this study is consistent with the absence of labelled cells in the cuneate and gracile nuclei.

Labelled neurons along the most ventral edge of the caudal brainstem, within the ventral spinocerebellar tract, could not be assigned to any previously described nucleus. Nudo and Masterton [57] found a group of spinally projecting cells in a similar location in the nine-banded armadillo, but not in the other 21 species they studied. They were unable to assign a name to this group of cells, but one possibility is that they represent a ventral extension of the lateral reticular nucleus, which has been shown to have neurons that project to the spinal cord [18].

In the G140 fetus there were only a few labelled cells per coronal section in the spinal trigeminal nucleus, whereas Nudo and Masterton [57] reported that the spinal trigeminal nucleus was a major source of projecting neurons in all 22 species they studied. This discrepancy is not explained by restriction of the CTB injection to the ventral horn in the G140 fetus, because the G55 fetus also showed only minor labelling in the spinal trigeminal nucleus. Nor does the spinal level of CTB injection in this study provide an explanation for the discrepancies found in spinal trigeminal labelling. Okado and Oppenheim [18] found spinal trigeminal labelling after retrograde tracer injections at lumbar regions in the chick, while Kudo et al. [25] found spinal trigeminal labelling after lower thoracic injections of retrograde tracer in the prenatal rat. These studies demonstrate that the projections from the spinal trigeminal nucleus reach levels caudal to C3-C4 and as such the projections should have picked up the CTB tracer used in our study. Thus the limited labelling of CTB cells in the spinal trigeminal nucleus suggests that the sheep has far fewer spinal projections from here than the species studied by Nudo and Masterton. In accord with this possibility Martin et al. [30] found only a few scattered cells in the spinal trigeminal complex after application of the retrograde tracer, Fast Blue, to the dorsal and ventral horns of the opossum spinal cord. Their finding, along with findings described herein, suggests there may be a distinct species difference in the extent of projections from the spinal trigeminal nucleus to the spinal cord; we have been able to find no plausible reason for such a difference.

The few labelled cells that formed a 'cap' over the inferior cerebellar peduncle in the G140 fetus have been left unnamed. Although they were found in all the sheep fetuses we studied, spinally-projecting neurons have not been reported in this location before. Based upon location, the cells could belong to either Nucleus Y or the infracerebellar nucleus, both of which lie above the inferior cerebellar peduncle [59]. However, neither of these nuclei has previously been shown to project axons to the spinal cord. Application of a retrograde tracer into the vestibular nucleus, which receives projections from nucleus Y [60], and in the oculomotor nucleus, which receives projections from the infracerebellar nucleus [61], would help to establish whether these labelled cells lie in either of these two nuclei. The other possibility is that the neurons that form the cap over the inferior cerebellar peduncle actually lie in the caudal pole of the vestibular nucleus which begins at this level [48]; however the vestibular nucleus has not been reported as extending over the dorsal surface of the inferior cerebellar peduncle in other species. The lack of CTB labelled cells in this area in the G55 fetus may mean that the neurons do not send projections to the spinal cord until a later developmental age, or alternatively, it may be that the nucleus in which they lie changes in position as development proceeds.

In the 22 species studied by Nudo and Masterton [57], retrogradely-labelled cells were found in the intercalated nucleus which lies between the hypoglossal nucleus and the dorsal motor nucleus of the vagus, and extends around the hypoglossal nucleus. Neither of these areas exhibited any CTB labelled cells in the fetal sheep brainstem at G140 or G55. A similar lack of retrogradely-labelled cells in the region surrounding the hypoglossal nucleus has been reported in the fetal rat [25], the chick [18] and opossum [30,62]. This discrepancy may be due to the fact that Nudo and Masterton [57] applied their tracer at C1-C2, a level considerably rostral to the positions of tracer injections in other reports [18,25,30,62], including the present study. Thus it is possible that the retrogradely-labelled cells observed around the hypoglossal nucleus by Nudo and Masterton [57] have projections that terminate at spinal levels C1-C2.

## Conclusion

This study has established that all major nerve fibre tracts between the brainstem and the spinal cord that have previously been identified in other species are present in the fetal sheep, including the reticulospinal, vestibulospinal, raphespinal and rubrospinal pathways. Our findings show that supraspinal inputs reach the spinal cord long before they exert an effect on spinally generated motor activity, suggesting that following their arrival, these projecting axons undergo a period of maturation before they can influence the spontaneous activity of the motor circuits of the spinal cord.

## Methods

### Surgery and CTB administration

This study used healthy Border-Leicester Merino cross ewes, with accurately time-dated pregnancies of 52 days (n = 4) or 135 days (n = 4). Maternal anaesthesia was induced with an intravenous injection of 40 ml Propofol (10 mg.ml^-1^; Diprivan 1% w/v, Zeneca Ltd. Macclesfield, United Kingdom) followed by intubation and subsequent maintenance of anaesthesia by inhalation of a halothane (1–2%), nitrous oxide (66%) and oxygen (32–33%) mixture.

The fetal head and neck were exposed via incisions through the maternal abdomen and uterus. An incision was made along the dorsal midline of the fetal neck between cervical levels C3-C6 to expose the vertebrae by dissecting away the overlying muscle. A laminectomy was performed to achieve a wide exposure of the dorsal surface of the spinal cord. The ewe was hyperventilated for a period of 1–2 min so that a period of maternal apnea lasting 60 s or more ensued when the ventilator was switched off. This ensured that the fetal spinal cord remained motionless for long enough for 0.5–1.0 μl of cholera toxin subunit B (CTB; Sapphire Bioscience, Alexandria, Australia) to be injected into the ventral horn of the gray matter. The method of injection of CTB was by hand using a SGE Hamilton syringe attached via a short piece of silicon tubing to a micropipette pulled from a glass capillary tube. A small amount of non-toxic blue dye was added to the CTB solution to allow visualisation of the injection site at postmortem. Once the injection was complete, the pipette remained in position for approximately 15 s to prevent the tracer leaking back up the injection tract before the micropipette was carefully withdrawn from the spinal cord and ventilation of the ewe resumed.

The skin of the fetal neck was closed with 3.0 silk sutures in the G135 fetuses and with 5.0 silk sutures in the G52 fetus. The fetus was returned to the uterus and between 500 -1000 ml of warm saline, containing 0.5 ml of broad spectrum antibiotic (Ilium Oxytet-200; Troy Laboratories, Smithfield, Australia; 200 mg.ml^-1^), was instilled into the amniotic sac to replace amniotic fluid lost during the operation. The uterine incision was carefully sutured to be watertight and the maternal abdomen and skin were sutured before a mesh stocking was positioned over the incision site for protection. The ewe received a 1 ml intramuscular injection of analgesic (Finadyne; Schering-Plough, Australia; 50 mg) and an intramuscular injection of 4.5 ml antibiotic (Ilium Oxytet-200; Troy Laboratories, Australia, 200 mg.ml^-1 ^oxytetracycline) before being returned to the animal house.

### CTB immunohistochemistry

Three to six days after the initial surgery, the ewe was again anaesthetised and the fetus was exposed through the original uterine incision. In the younger fetuses we removed a portion of the thoracic wall on the left side of the fetus to expose the descending aorta. A teflon catheter (OD 0.07 cm: ID 0.04 cm) was inserted into the vessel and heparinised saline warmed to 40°C was infused through it until all blood was cleared from the fetal circulation. To assist clearance the right atrium was widely incised. A similar procedure was performed in the older fetal group except that their large size made it a simple matter to insert the teflon catheter (OD 0.07 cm: ID 0.04 cm) into the femoral artery. Once blood washout was complete, as judged by complete clearance from the jugular veins and tongue, a fixative solution containing 4% formaldehyde plus 15% saturated picric acid in 0.1 M phosphate buffer at a pH of 7.4 was infused through the fetal circulation until the tissues of the head had become a yellow colour and the jaw had become rigid; this required up to 1000 ml for the larger fetuses and a lesser volume in the smaller fetuses. The ewe was killed with an overdose of anaesthetic immediately after the fixation began (20 ml Lethabarb, pentobarbitone sodium; Virbac, Sydney, Australia).

Sectioning of the fetal brainstem was performed as described in Stockx et al., 2007 [48]. Briefly, the CNS of the fetus was dissected free and stored in fixative overnight at 4°C, followed by cryoprotection, by overnight immersion in 0.1 M phosphate buffer (pH 7.4) containing 20% sucrose. The cerebral hemispheres were removed from both the G140 and G55 fetuses. Once the cerebellum was removed from the G140 fetuses (only a small rudiment existed in the younger fetuses) the brainstems of both the G55 and G140 fetus were laid ventral side down on a horizontal surface. Cuts were made in the coronal plane of the brainstem: at the obex, just caudal to the cerebral peduncles, immediately rostral to the cerebral peduncles and, through the pulvinar and habenula nuclei, rostral to the level of the pineal gland. The brainstem segments were placed on their caudal cut surfaces on the platform of a freezing microtome (G140) and frozen with liquid carbon dioxide, or placed into cryomoulds (G55) and frozen with liquid nitrogen for sectioning on a cryostat. Sections 50 μm thick were cut parallel to this plane. Every 6^th ^section was collected and either mounted onto gelatin-coated slides or left free floating for visualisation of CTB immunoreactivity.

Free-floating and slide-mounted sections were incubated overnight in primary antibody goat-anti CTB serum (1:5000; Sapphire Bioscience, Alexandria, Australia) diluted in phosphate buffer saline (PBS; 0.1 M, pH 7.4). The following day the brainstem sections were washed 3 times, each for 10 min in PBS, followed by incubation at room temperature in biotinylated donkey anti-sheep IgG (H + L) serum (MDA Pharma, NSW, Australia; 1:200 dilution). The sections remained in the serum for 1 hour before being given three 10 min washes in PBS. Sections were then incubated with streptavidin-biotinylated horseradish peroxidase complex (Amersham Australia, NSW; 1:100 dilution) for 1–2 hrs before they were reacted with 3,3'-diaminobenzidine tetrahydrochloride (DAB; Sigma Aldrich, NSW, Australia). All free floating sections were then mounted onto gelatin-coated slides, and all sections, including those that were mounted prior to immunohistochemical labelling for CTB, were dehydrated and cover-slipped using DPX.

Brainstem sections were viewed under a Leitz 22 light microscope to identify all nuclei containing CTB labelled neurons. Nuclei were identified on the basis of a fetal sheep atlas [48]. Figures were prepared in Adobe Photoshop Elements (version 2) either from images scanned directly from the slide using a Nikon scanner (Coolscan 50) with a FH-GI attachment, or from electronically stored images obtained using a digital camera connected to a Zeiss microscope. An outline of the left hand side of the figure was made digitally and the position of CTB labelled neurons marked on this digital copy. This image was then flipped horizontally so the original section could be visualised facing the digital section. Contrast levels and the brightness of figures were modified to enhance the CTB labelled cells. In a few instances erythrocytes remaining in blood vessels reacted to the DAB procedure and in such cases large blood vessels were deleted from the image using the cloning tool to allow clearer visualization of the CTB labelled cells. No other digital modifications were made to the images.

## Authors' contributions

EMS, PJB, CRA, SMM and IRC were all involved in the conception and design of this study. All authors were involved in the preparation of this manuscript. EMS and PJB performed the surgical procedures, EMS and SMM performed the histochemistry studies, EMS and CRA analysed the data. Figures were prepared by EMS.
